# A Comparative Assessment of Chromium–Boron Hardfacing Using SMAW and FCAW Techniques

**DOI:** 10.1155/2024/4943983

**Published:** 2024-10-28

**Authors:** Chowda Reddy C., K. M. Kenchi Reddy, C. T. Jayadeva, Ramesh Kumar S. C., R. Vara Prasad Kaviti, Abhijit Bhowmik, Chander Prakash

**Affiliations:** ^1^Department of Mechanical Engineering, Adichunchanagiri Institute of Technology, Chikkamagaluru, Karnataka, India; ^2^Department of Mechanical Engineering, Srikrishna Institute of Technology, Bengaluru, Karnataka, India; ^3^School of Mechanical Engineering, Reva University, Bengaluru, Karnataka, India; ^4^Department of Mechanical Engineering, Brindavan College of Engineering, Bengaluru, Karnataka, India; ^5^Department of Mechanical Engineering, Dream Institute of Technology, Kolkata 700104, India; ^6^Centre for Research Impact & Outcome, Chitkara University Institute of Engineering and Technology, Chitkara University Rajpura 140401, Punjab, India; ^7^University Centre for Research and Development, Chandigarh University, Mohali, Punjab 140413, India

**Keywords:** FCAW, hardfacing, microstructure, SMAW

## Abstract

This research paper investigates the effectiveness of shielded metal arc welding (SMAW) and flux-cored arc welding (FCAW) on mild steel substrates for chromium–boron hardfacing. Chromium–boron alloys are hard-wearing and corrosion-resistant materials used in industries where wear resistance is critical. The study aims to identify the best welding technique for increasing surface hardness and wear resistance. Standard test specimens were chosen and deposited using SMAW and FCAW processes. SMAW uses an electrode covered with flux, which turns into a sticky state when heated, while FCAW uses a core wire fabricated from flux, which generates a shielded gas upon melting. The effectiveness of each welding technique is assessed based on deposition efficiency, dilution rate, microstructure, hardness distribution, and wear resistance. This research helps industries choose the most efficient material and method for improving wear and corrosion resistance in applications like mining, construction, agriculture, and manufacturing. On average, FCAW offers a 1.67% improvement in hardness and 28.12% improvement in mass loss reduction when compared to SMAW.

## 1. Introduction

In industrial sectors where machinery and equipment are subjected to severe wear and abrasion, the concept of hardfacing emerges as a crucial solution to extend the service life and performance of critical components. Hardfacing involves the application of a wear-resistant layer onto the surface of engineering parts, effectively shielding them from the detrimental effects of friction, erosion, and corrosion [1–3]. The significance of hardfacing lies in its ability to enhance the durability and reliability of components without necessitating a complete replacement, thereby reducing downtime and maintenance costs. By overlaying the base material with a layer of hardfacing alloy, engineers can tailor the surface properties to withstand specific operational challenges, ensuring prolonged operational efficiency even in the harshest of environments [4].

Various materials can be used for hardfacing, ranging from traditional metals like chromium, nickel, and cobalt to more advanced carbides, borides, and complex alloys. Each material offers distinct advantages in terms of hardness, wear resistance, and chemical stability, allowing for customization based on the specific requirements of the application [5]. The effectiveness of hardfacing processes heavily depends on the welding technique employed. SMAW and FCAW are two widely used methods for applying hardfacing alloys due to their versatility and suitability for various work environments. SMAW, often referred to as stick welding, employs a consumable electrode coated in flux to shield the weld pool, while FCAW employs a continuously fed tubular electrode with a flux core [6].

This study aims to provide a comparative assessment of chromium–boron hardfacing applied using SMAW and FCAW techniques. By systematically evaluating the performance of these two welding processes, this research seeks to determine their respective advantages, limitations, and suitability for specific industrial applications. The comparative assessment will involve analyzing various parameters such as deposition efficiency, dilution rate, microstructure, hardness distribution, and wear resistance of the resulting hardfaced surfaces. Understanding how SMAW and FCAW influence these key factors will provide valuable insights into the optimal selection of welding methods for chromium–boron hardfacing applications [7]. Furthermore, the findings of this study will contribute to the body of knowledge surrounding hardfacing processes, aiding industries in making informed decisions regarding material selection, process optimization, and performance enhancement. Such insights are particularly crucial for sectors where wear and corrosion resistance are paramount, including mining, construction, agriculture, and manufacturing [8].

Hardfacing processes which find their application in metallurgy and material sciences are used for the improvement in surface characteristics of engineering parts. Chromium–boron alloys are fairly popular and renowned for their hardness and wearing quality resistance to corrosion. They can be used via SMAW and FCAW, and the two have their benefits and drawbacks All in all, we can apply these materials via SMAW and FCAW, with the former being more advisable than the latter [9–11]. SMAW employs a consumable electrode which is coated in flux so that it lays down the hardfacing material to the substrate while the flux melts and disappears to produce a protective gas and a slag. This method is favorable on the basis of cost and versatility but proves to be time-consuming significantly. FCAW employs a consumable tubular wire containing flux, and when melted they produce a shielded gas. This method has advantages of high deposition rates, increase in productivity, and enhanced control of the welded joints. This comparative analysis seeks to draw a comparison between SMAW and FCAW processes, regarding their efficiency in applying chromium–boron hardfacing in order to determine the efficiency of welding types for certain industries [12–14]. For this study, mild steel is used as the base due to its common usage in applications where the material's wear resistance is needed. The material used for the hardfacing is chromium–boron alloy which has a very high hardness and its abrasive characteristics are well known. The plates were prepared as substrates and an appropriate cleaning afterward was performed to allow for the best adhesion. The chromium–boron alloy was apparently impressed using SMAW as well as FCAW [15, 16]. The objective is to analyze which of the two methods is more suitable for enhancing surface hardness and wear resistance.

**Figure 1:**
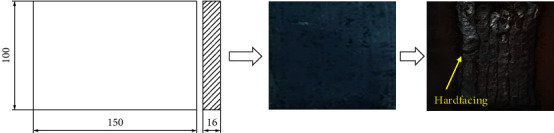
Standard test specimens.

We prepare standard test specimens as shown in Figure 1. The process includes selecting the appropriate material, procuring the necessary plates or sheets, cutting the specimens, cleaning the surfaces, grinding if necessary, deburring sharp edges, and roughening the surface if necessary. The specimens are stored in a clean and dry environment to prevent contamination or corrosion. By following these steps, accurate and reliable test results can be achieved, aiding in the selection of hardfacing alloys for applications demanding high abrasion and wear resistance. Chromium–boron alloy electrodes (such as VAUTID 143) have proven to be an excellent choice [17, 18].

The studies encompassing the Global Welding Trends of high-strength and stainless steels and welding technologies, including SMAW, FCAW, Gas Metal Arc Welding (GMAW) and Submerged Arc Welding (SAW), and their general performance characteristics. Farneze et al. conducted a comparison between SMAW and FCAW weld metals designed for offshore applications and mooring chains, focusing on the mechanical properties of high-strength steel (HSS) welds, particularly their tensile strength. Similarly, Surian et al. explored SMAW, FCAW, and SAW deposits, specifically addressing the challenges related to tensile properties in high-strength ferritic materials, which are crucial for load-bearing applications [19, 20].

In line with this study, Ahmad and others in a study done in the year 2024 sought to establish the influence of impact toughness on SMAW, FCAW, and GMAW on API X60 pipeline steel and noted that a variety of welding processes result in variation in toughness and mechanical performance. This is important for the pipeline's performance since flexibility implies impact resistance. Silveira et al. provided insights into the corrosion resistance of multipass welding on lean duplex stainless steel UNS S32304, using SMAW, FCAW, and GMAW techniques. Their research focuses on the corrosion resistance and mechanical performance of thick-walled plates after welding, intended for use in harsh environments [20–22].

Welded failure modes in AISI/SAE 304 stainless steels were described by Galvis and Hormaza [23]. This study demonstrates the critical impact of selecting the appropriate welding process on material performance and failure resistance. The choice of welding method directly influences the serviceability and durability of the material. Together these references emphasize the importance of determining right strategies for welding taking into consideration mechanical characteristics, corrosion resistance, and failure patterns especially in HSLA and stainless steels in offshore, pipeline, and construction fields [23].

Current works highlight the significant development in the application of hardfacing procedures such as SMAW and FCAW specifically designed to enhance the wear and corrosion resistance in severe industrial conditions. Chromium–boron alloys are rapidly gaining appreciation for their hardness, wear, and corrosion resistance, owing to their uses in mining, construction, and agriculture. Research analysis reveals that FCAW provides improved arc stability, metal deposition efficiency, and microstructural refinement than SMAW, thus improving the surface characteristics. Despite its drawbacks, SMAW remains popular due to its simplicity and cost-effectiveness. Conducting a comparative analysis is crucial for industries to evaluate the advantages and disadvantages of these welding techniques, enabling them to choose the most suitable method for their specific requirements.

## 2. Materials and Methods

### 2.1. Materials

The base material utilized is medium carbon steel, selected due to its prevalent use in applications that require enhanced wear resistance. Because of its superior structural integrity, machinability, and weldability, ASTM A36 mild steel is an affordable, adaptable substrate material that can be used for hardfacing alloy applications [24]. The chemical composition of mild steel is shown in Table 1.

### 2.2. Sample Preparation

The chemical composition of the electrodes is as per Table 2.

### 2.3. Welding Details and Parameters

**Figure 2:**
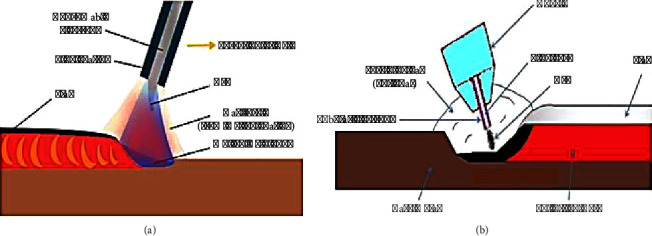
Welding process. (a) SMAW. (b) FCAW.

The welding process for chromium–boron hardfacing alloy on mild steel ASTM A36 substrates involves two processes: SMAW and FCAW conducted at Arya Technocrats, Belgaum, Karnataka, as seen in Figures 2(a) and 2(b), respectively.

Table 3 shows SMAW and FCAW process parameters, and the study progressed welding through three stages by varying welding parameters like current, travel speed, and voltage.

Table 4 shows that samples A1 and B1 account for 6.69% of the total heat input, whereas samples A3 and B3 contribute 8.51%. Samples A4 and B4 exhibit a higher heat input percentage (8.45%) than samples A5 and B5 (7.60%) and A6 and B6 (6.91%) because of their increased travel speeds. The examination reveals an equal spread of heat input percentages between SMAW and FCAW methods, demonstrating a uniform approach to applying heat input. These data are essential for maximizing welding settings in hardfacing welding procedures.

### 2.4. Characterization

#### 2.4.1. Microstructural Characterization

The process involves preparing hardfaced mild steel samples, grinding and polishing them, etching them, examining their microstructure using optical or SEM microscopes, and analyzing their features using image analysis software to determine the impact of welding processes on the steel properties [25]. We document and interpret the observed microstructures to gain insights into the material properties and the effectiveness of the hardfacing process.

#### 2.4.2. Mechanical Characterization

The dry sand abrasive wear test is a standardized method used to evaluate the resistance of materials to abrasive wear under dry conditions. The test involves sample preparation, selection of abrasive material, test setup, application of abrasive material, measurement of wear, control of test parameters, and data analysis and interpretation. The test allows for the measurement of wear and material loss and the calculation of wear rate or resistance [26, 27].

### 2.5. Wear Test Conditions

The importance of incorporating wear test conditions in the comparative evaluation lies in accurately assessing material performance. The tests adhere to the ASTM G65 standard and replicate abrasive wear conditions seen in industrial settings. Silica sand, with a particle size ranging from 212 to 300 *μ*m, is the abrasive medium utilized. The samples are made as per dimensions and uniform surface texture. Room temperature and relative humidity are kept at about 50% to maintain environmental conditions. The all-encompassing method boosts the strength of the comparison evaluation by offering a precise and repeatable process for assessing the wear resistance of chromium–boron hardfacing materials under actual operating conditions.

## 3. Results and Discussion

### 3.1. Microstructure Examination

The microstructure of hardfaced mild steel samples was observed using SEM. Results showed that welding current significantly impacted the microstructure. Low currents led to incomplete fusion and poor penetration, while optimal currents resulted in well-defined fusion lines and uniform distribution of hard phases. High currents led to overfusion and potential overheating. Constant travel speed ensured consistent heat input and weld bead geometry across all samples. The interface between the hardfacing alloy and substrate showed good metallurgical bonding, with no visible defects or discontinuities. SEM examination provides detailed insights into microstructural features, allowing for precise evaluation of welding parameters' influence on material properties [28, 29].

**Figure 3:**
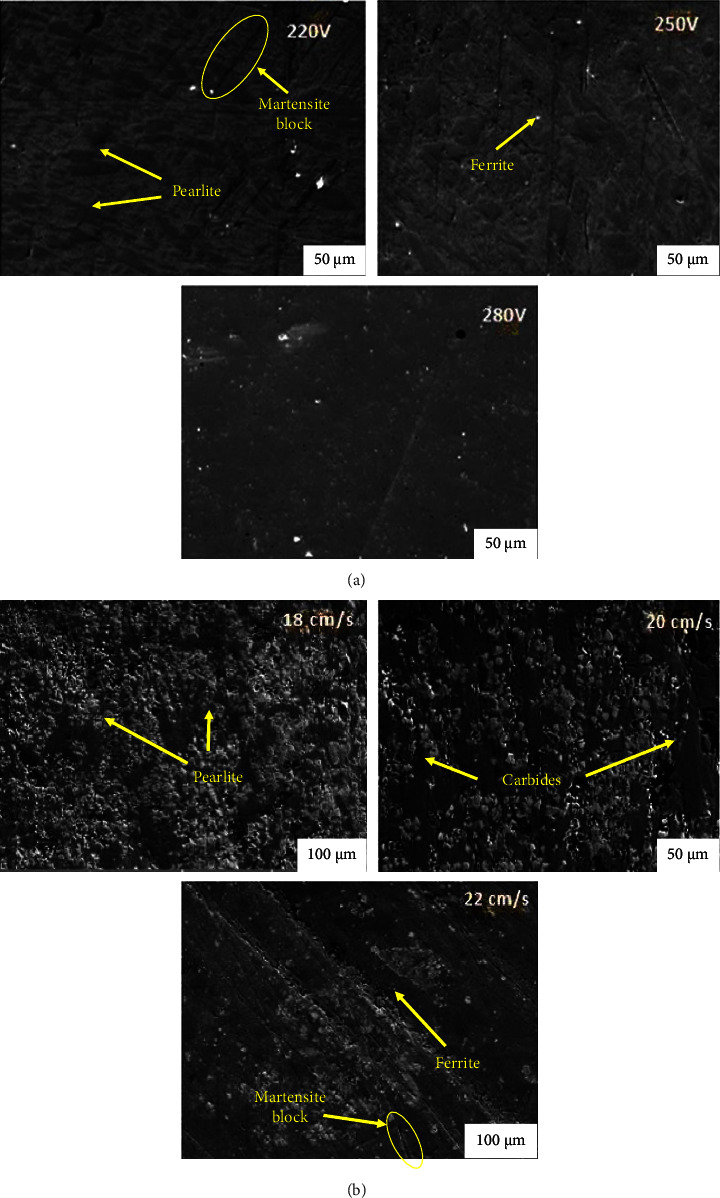
SEM micrograph of SMAW by varying current and travel speed. (a) Varying current and constant travel speed. (b) Varying travel speed and constant current.

**Figure 4:**
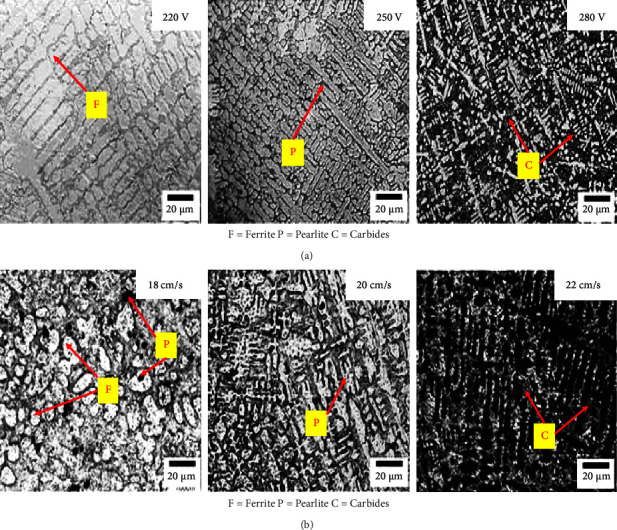
SEM micrograph of FCAW by varying current and travel speed. (a) Varying current and constant travel speed. (b) Varying travel speed and constant current.

SEM micrographs from SMAW shown in Figures 3(a) and 3(b) and FCAW revealed in Figures 4(a) and 4(b) show varying characteristics with varying current and constant travel speed and vice versa, respectively [30]. At lower currents, they may show incomplete fusion, irregular microstructure, inadequate heat input, and overheating. At optimal currents, they reveal well-defined fusion lines and uniform distribution of hard phases. At higher currents, overheating may be observed, with coarse grain structure and large carbides. At constant current and travel speed, they may show excessive heat input, wide weld beads, and insufficient heat input. At constant current and travel speed, they may show narrower weld beads, incomplete fusion, and lack of penetration [31–33].

### 3.2. Hardness Test

**Figure 5:**
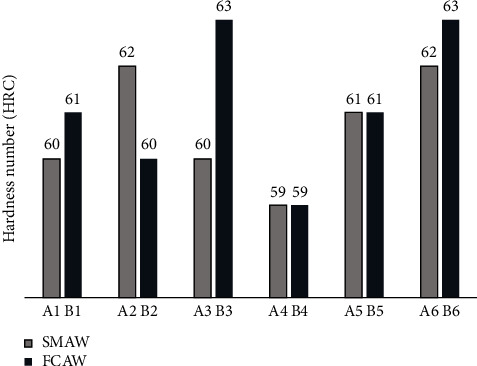
Rockwell hardness values for SMAW and FCAW.

Orthogonal array samples with HRC values show that higher hardness generally corresponds to enhanced strength and wear resistance, while lower hardness values may suggest reduced mechanical properties. By comparing hardness values from different welding parameters, it is possible to identify trends and correlations between welding conditions and material hardness as shown in Figure 5, aiding in process optimization and quality control [34–37].

Hardness is crucial in welding, as it indicates a material's strength and wear resistance, impacting the performance and durability of welded structures. Factors like current, voltage, and travel speed influence hardness significantly. Higher current results in more heat input, creating a larger weld pool and deeper fusion, while lower current may lead to inadequate fusion. Faster travel speeds reduce heat input, resulting in quicker cooling and heightened hardness. Increased voltage improves stability and grain growth [38–41]. Analyzing welding conditions and hardness levels aids in establishing optimal parameters for high-quality welds. Managing hardness values through welding parameters is essential for superior weld production. Percentage difference of FCAW offers a 1.67% improvement in hardness compared to SMAW.

### 3.3. Wear Test

**Figure 6:**
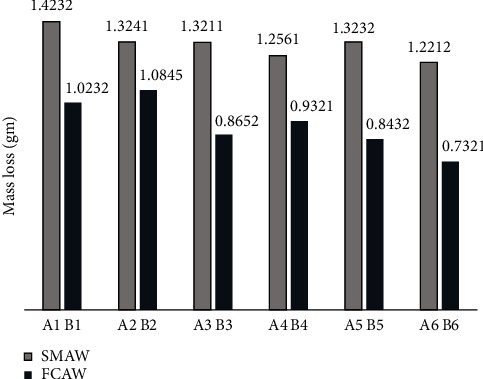
Mass loss of the hardfaced samples.

Dry sand abrasive wear tests are typically conducted to evaluate the wear resistance of materials under abrasive conditions. The material is subjected to abrasive particles (sand) under a specific load of 130 N and a rotational speed of 200 rpm. This test allows for the calculation of wear rate or resistance, the quantification of material loss, and the evaluation of which material is best suited for the intended application. The results can then be compared to those of alternative materials, surface treatments, or coating formulations [42–48]. Based on the study, we can conclude that FCAW is the best technique since it tends to have the lowest mass loss when compared to the other as shown in Figure 6. The study reveals that FCAW has demonstrated a 28.12% improvement over SMAW in this specific context.

## 4. Discussion

This article provides a comparative assessment of chromium–boron hardfacing using SMAW and FCAW techniques. Chromium–boron hardfacing alloys are known for their excellent wear resistance and ability to withstand high temperatures, making them suitable for applications such as earthmoving equipment, mining equipment, extrusion dies, and other parts exposed to high abrasion, impact, and sliding wear. SMAW is a popular welding technique used for hardfacing, which involves the use of a consumable electrode coated with a flux that provides shielding gas, slag, and deoxidizers. It is manual, making it more time-consuming than other welding techniques but providing excellent control over the welding process, resulting in good weld quality and low porosity. FCAW, on the other hand, is a semiautomatic or automatic welding technique that uses a continuously fed electrode with a hollow core filled with flux. It provides a higher deposition rate and faster welding speed than SMAW, making it ideal for welding thicker sections and providing better penetration and fusion than SMAW.

Both SMAW and FCAW are suitable for chromium–boron hardfacing applications, but there are some differences in weld quality, welding speed, and equipment costs. SMAW provides better control over the welding process, resulting in good weld quality and low porosity. FCAW provides faster welding speed and higher deposition rate, reducing overall welding time and labor costs. In inference, chromium–boron hardfacing offers excellent wear resistance and durability for various industrial applications. Both SMAW and FCAW techniques are suitable for chromium–boron hardfacing, but each has its advantages and disadvantages. The choice between SMAW and FCAW depends on specific application requirements, welding skills, and budget constraints. A comparative assessment of these techniques provides valuable insights into their capabilities, enabling informed decisions in selecting the appropriate hardfacing technique for specific industrial applications.

## 5. Conclusions

The comparative assessment of SMAW and FCAW techniques for chromium–boron hardfacing reveals key findings.

• The study found that FCAW has higher deposition efficiency than SMAW due to continuous wire feed and higher deposition rates, resulting in faster hardfacing layer buildup. It also showed higher welding productivity due to its semiautomatic nature and higher deposition rates.
• Both SMAW and FCAW techniques produced satisfactory welds in terms of bead appearance, fusion, and penetration, but careful parameter optimization is required to minimize defects.
• Hardness and wear resistance tests showed comparable results between SMAW and FCAW specimens, meeting the requirements for abrasion and erosion protection in industrial applications. However, FCAW may involve higher equipment and consumable costs compared to SMAW.
• The cost-effectiveness of SMAW or FCAW for chromium–boron hardfacing depends on factors like material wastage, labor expenses, and project scale. The choice should be based on project requirements, budget constraints, and available resources, with SMAW being a reliable method.
• FCAW provides advantages in terms of productivity and deposition efficiency, making it suitable for high-volume or automated hardfacing operations. Further research and experimentation may be beneficial to optimize process parameters and enhance the performance of chromium–boron hardfacing using these welding techniques.

## Figures and Tables

**Table 1:** Chemical composition of mild steel ASTM A36 as base metal (wt.%).

Element	C	Cu	Fe	Mn	P	Si	S
Content	0.27	0.20	98.0	1.03	0.040	0.280	0.050

**Table 2:** Chemical composition of hardfacing electrode (wt.%).

Element	Cr	C	Si	B	Fe
Content	19.97	2.35	0.87	1.89	Bal

**Table 3:** SMAW and FCAW process parameters.

	SMAW	FCAW
Input power	220–280 V, 50/60 Hz	220–280 V, 50 Hz
Welding AMP range	200–300 A	200–3000 A
Open circuit voltage	60 VDC	80–90 VDC
Travel speed	18–22 cm/min	18–22 cm/min

**Table 4:** The Taguchi L9 orthogonal array was used for varying current and travel speed across six trials.

Hardfacing welding process		Current (amp)	Travel speed (cm/min)	Heat input	Total input
SMAW	FCAW			*Q* (J/cm)	Percentage (%)
A1	B1	220	20	79.2 × 10^3^	6.69
A2	B2	250	20	90 × 10^3^	7.60
A3	B3	280	20	100.8 × 10^3^	8.51
A4	B4	250	18	100 × 10^3^	8.45
A5	B5	250	20	90 × 10^3^	7.90
A6	B6	250	22	81.81 × 10^3^	6.91

## Data Availability

The data that support the findings of this study are available from the corresponding authors upon reasonable request.
